# Mean Six Minute Walk Distance of Healthy Healthcare Workers of a Tertiary Care Centre: A Descriptive Cross-sectional Study

**DOI:** 10.31729/jnma.7541

**Published:** 2022-07-31

**Authors:** Subash Pant, Krity Basnet, Prinsa Shrestha, Mathura K.C.

**Affiliations:** 1 Department of Internal Medicine, Kathmandu Medical College and Teaching Hospital, Sinamangal, Kathmandu, Nepal; 2 Kathmandu Medical College and Teaching Hospital, Sinamangal, Kathmandu, Nepal

**Keywords:** *exercise test*, *health personnel*, *healthy volunteer*, *walk test*

## Abstract

**Introduction:**

The six-minute walk test is a sub-maximal exercise test used in clinical populations to determine functional exercise capacity. It is a safe, simple, and inexpensive test. There are a number of reference equations described for estimating six-minute walk distance in healthy subjects in different countries. However, there is a lack of standard reference value for six minute walk distance in healthy Nepalese population. The aim of the study was to find the mean six minute walk distance of healthy healthcare workers of a tertiary care centre.

**Methods:**

A descriptive cross-sectional study was conducted among healthy health care workers of a tertiary care centre from 1 August 2021 to 30 November 2021 after taking ethical approval from Institutional Review Committee (Reference number: 1507202105). Convenience sampling was done. Point estimate and 95% Confidence Interval were calculated.

**Results:**

The mean six-minute walk distance of the 162 healthy health care workers was 486.74±74.73 (475.23-498.24, 95% Confidence Interval) m. Men walked 519.61±79.19 m and women walked 474.12±75.62 m. The mean age of the participants was 29.25±8.25 years.

**Conclusions:**

The mean six-minute walk distance was found to be lower when compared to similar studies conducted in similar settings.

## INTRODUCTION

The six-minute walk test (6MWT) is a sub-maximal exercise test used to determine functional exercise capacity.^1^ It measures the distance travelled within a period of six minutes. It is a safe, simple, and inexpensive test and an important component of quality of life, because it indicates the ability to carry out daily tasks.^1,2^ Several disorders, such as obstructive pulmonary disease, heart failure, arthritis, and neuromuscular disease, diminish the six-minute walk distance (6MWD).^1^ The American Thoracic Society has developed guidelines for the 6MWT in clinical settings.^1^

There are a number of reference equations described for estimating 6MWD in healthy subjects in different countries.^3-12^ However, there are no studies to find out the reference range of 6MWT in healthy Nepalese population yet.

The aim of this study was to find out the mean value of 6MWD in health workers of a tertiary care centre.

## METHODS

A descriptive cross-sectional study was done in Kathmandu Medical College and Teaching Hospital (KMCTH), Kathmandu, Nepal from 1 August 2021 to 30 November 2021. Ethical approval was taken from the Institutional Review Committee of KMCTH (Reference number: 1507202105). Healthcare workers of >18 years and <65 years working in KMCTH who provided consent for the study were included in the study. Smokers, individuals with history of any chronic morbidities, history of respiratory infection within four weeks before the recruitment, abnormal pulmonary function were excluded from the study. Convenience sampling was done, and sample size was calculated using the formula:


$$\begin{array}{ll} \text{n} & ={\text{Z}}^{2}\times \frac{\sigma^{2}}{{\text{e}}^{\text{2}}}\\ & =1.96^{2}\times \frac{55.51^{2}}{9.00^{2}}\\ & =147 \end{array}$$

Where,

n = minimum required sample size
Z = 1.96 at 95% Confidence Interval (CI)
σ = Standard deviation taken from previous study^8^
e = margin of error, 9.00

The calculated sample size was 147. However, 162 samples were taken.

The 6MWT was conducted according to a systematised protocol.^1^ The normal pulmonary function was defined as Forced Expiratory Volume in 1 second (FEV1)>80% predicted, and FEV1 to Forced Vital Capacity (FVC) ratio >0.7 according to the European Respiratory Society (ERS)/American Thoracic Society (ATS) guideline.^13,14^ Dyspnea was measured with the modified Borg dyspnea scale.^15^ Oxygen saturation (SpO_2_), and pulse rate (PR) were assessed at the start of the six minute walk test, at an interval of 1 minute during the test, and at the end of the test. The 6MWT was done twice; two tests were performed on the same day and separated by 20 minutes.

IBM SPSS Statistics version 24.0 was used for data entry and analysis. Point estimate and 95% Confidence Interval were calculated.

## RESULTS

The mean 6MWD of the 162 healthy health care workers was 486.74±74.73 (475.23-498.24, 95% CI) metres. The mean distance for men was 519.61±79.19 metres and for women was 474.12±75.62 metres. The parameters of the first and second six-minute walk test are tabulated below (Table 1).

**Table 1:** Parameters of first and second 6MWT (n = 162).

	First 6MWT Mean±SD	Second 6MWT Mean±SD
Distance (metre)	486.71±81.09	486.74±74.73
Sex		
Males	515.77±86.48	519.61±79.19
Females	474.12±75.62	472.49±68.28
Vitals		
Baseline PR (bpm)	84.12±11.08	85.35±9.82
Maximum PR (bpm)	110.96±12.11	111.43±11.54
Baseline SpO_2_ (%)	97.94±1.10	97.85±1.167
Lowest SpO_2_ (%)	94.52±1.81	94.85±1.70

There were 49 (30.24%) males and 113 (69.75%) females. The mean age of the participants was 29.25±8.25 years. The mean Body Mass Index (BMI) was 24.29±3.85 kg/m^2^ (Table 2).

**Table 2:** Baseline characteristics of the participants (n = 162).

Characteristics	Mean±SD
Age	29.25±8.25
Weight (kg)	61.31±11.09
Height (metres)	1.58±0.09
BMI (kg/m^2^)	24.29±3.85
FEV1 (litres)	2.79±0.62
FEV1% predicted	100.48±15.27
FVC (litres)	3.87±8.03
FVC% predicted	101.67±14.99
FEV1/FVC	0.98±0.12

Among the participants149 (91.97%) had an activelifestyle whereas 13 (8.24%) had a sedentary lifestyle. Most of the participants belonged to the age group 25-34 years (Table 3).

**Table 3:** Age distribution of the participants (n = 162).

Age range (years)	n (%)
18-24	49 (30.24)
25-34	81 (50)
35-44	20 (12.34)
45-54	9 (5.55)
55-64	3 (1.85)

Using the Borg scale,the degree of dyspnea wasclassified which is tabulated below (Table 4).

**Table 4:** Degree of dyspnea using Borg scale (n = 162).

	First 6MWT n (%)	Second 6MWT n (%)
Pretest Borg scale of 1	162 (100)	162 (100)
End Borg scale 2	79 (48.80)	80 (49.40)
End Borg scale 3	65 (40.10)	56 (34.60)
End Borg scale 4	13 (8) 21 (130)
End Borg scale 5	5 (3.10)	3 (1.90)
End Borg scale 6	-	2 (1.23)

## DISCUSSION

The 6MWT is a widely used test for objectively assessing functional exercise capacity in individuals with moderate-to-severe pulmonary illness. Unlike pulmonary function testing, the 6MWT detects the extrapulmonary symptoms of chronic respiratory disease, such as cardiovascular disease, frailty, sarcopenia, and malignancy, which often overlap. Unlike cardiopulmonary exercise stress testing, this test does not necessitate the use of complicated equipment or technical knowledge.^16^ The 6MWT is useful for assessing functional exercise capacity, determining prognosis, and assessing therapy response in a variety of respiratory disorders.^17^ The 6MWD reflects the functional exercise level for everyday physical activities because most activities of daily living are undertaken at submaximal levels of exertion.^1^

In healthy Caucasian people, regression equations have previously been published as a standard reference for 6-min walk distance (6MWD). However, a recent study discovered that the normal predicted 6MWD varies by ethnicity.^12^

In healthy subjects, the 6MWD ranges from 400 to 700m, the main predictor variables being gender, age and height.^3-12^ Six-minute walk distance is shown to vary according to ethnicity in different studies in the published literature. The mean 6MWD in study conducted in Spain was 571±90 m,^5^ another study was 614±56 m,^6^ New York was 698±96 m,^7^ China was 601.6 m,^8^ in another study at China was 627.3±52.88 m,^9^ similarly in another study was 659±62 m,^10^ in Korea was 598.5±57.92 m,^11^ and in Saudi Arabia was 409±51.^12^ The median 6MWD was 576 m in study conducted in USA.^4^ The mean 6MWD of our study was found to be lesser compared to the published literature. This might be because 6MWD varies significantly with height and gender.^1^ Since, our subjects included comparatively more females, the mean 6MWD was found to be lesser. Furthermore, the height of the Nepalese population is lesser compared to the western population in whom most of the previous studies were conducted. This might have contributed to lesser mean 6MWD of our subjects compared to published studies.

The mean 6MWD of males (519.61±79.19) was found to be more than that of females (474.12±75.62) m in our study. This is in agreement with the previous studies which have shown that the 6MWD of males is significantly greater than that of females.^3-12^

In addition to age, gender, and height, we explored other possible factors that could influence the results of the 6MWD, such as the post-walk Borg dyspnoea score. Although assessing the post-walk Borg dyspnoea score has been recommended by the ATS guidelines,^1^ only two studies have reported its value after the test.^5,6^ They did not observe any influence of dyspnoea on the 6MWD, and this finding is supported by our results.

There are some limitations in our study. The individuals older than 65 years of age were excluded from our study. The 6MWT has clinical utility for several diseases, such as cardio-respiratory disease, which often occurs in the elderly population, and our reference values are not applicable to this population. In the current study, the subjects were medical personnel and workers at a tertiary care centre. So, they may not be representative of the entire Nepalese population. Thus, the results cannot be generalised to the whole population.

## CONCLUSIONS

The mean six-minute walk distance was found to be lower when compared to similar studies conducted in similar settings. It is recommended to conduct a national multi-center study in a wide age range of healthy volunteers to establish the reference value of 6MWD for the Nepalese population. Further studies need to be performed to investigate the influence of demographics, anthropometrics, and habitual exercise activity on the 6MWD.
